# Supplementary figures and images for: Phylogenetic Analysis of the Complete Mitochondrial Genomes in the Ten Rupicapra Subspecies and Implications for the Existence of Multiple Glacial Refugia in Europe
Source: Animals (Basel). 2022 Jun 1;12(11):1430. doi: 10.3390/ani12111430 (PMC9179332; doi:10.3390/ani12111430)

Figure S1\_NJ tree

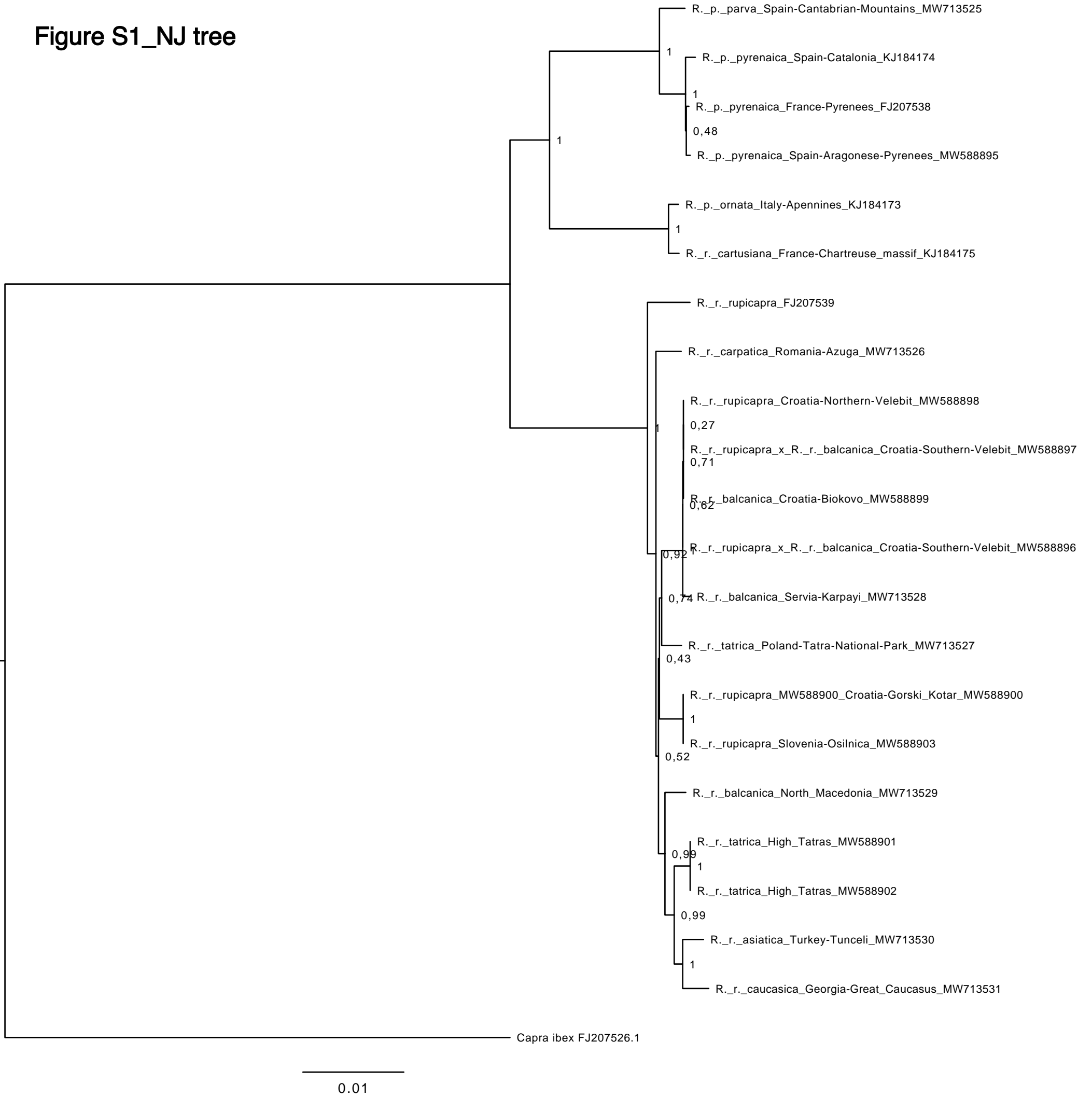

## Figure S2\_MP tree

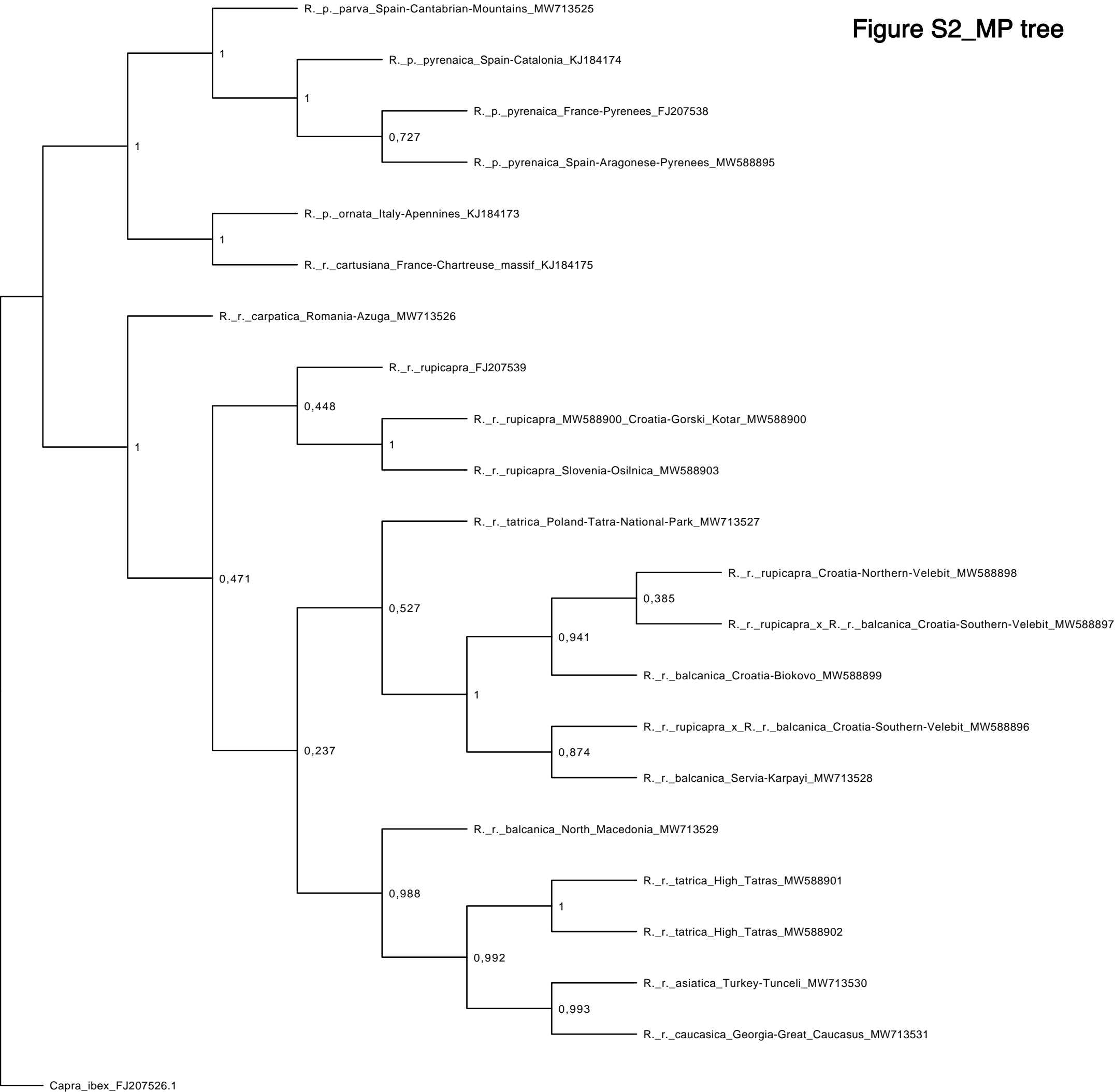

Figure S3\_ML tree

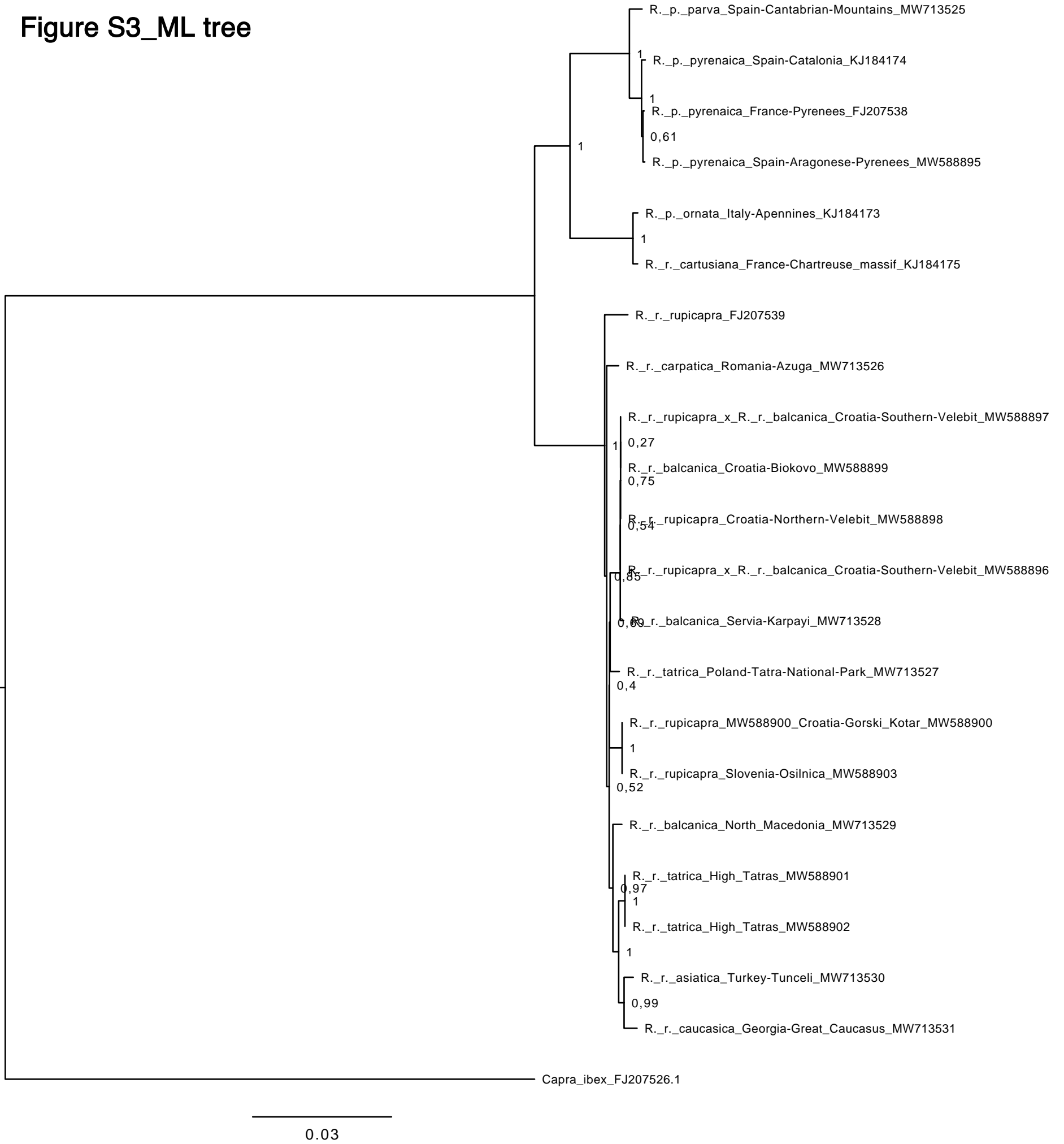

Supplement: Supplementary file 1 [file animals-12-01430-s001.zip › animals-1734472-supplementary.pdf]
